# Prediction of STN-DBS for Parkinson’s disease by uric acid-related brain function connectivity: A machine learning study based on resting state function MRI

**DOI:** 10.3389/fnagi.2023.1105107

**Published:** 2023-02-07

**Authors:** Bowen Chang, Chi Xiong, Chen Ni, Peng Chen, Manli Jiang, Jiaming Mei, Chaoshi Niu

**Affiliations:** ^1^Department of Neurosurgery, The First Affiliated Hospital of USTC, Division of Life Sciences and Medicine, University of Science and Technology of China, Hefei, Anhui, China; ^2^Anhui Province Key Laboratory of Brain Function and Brain Disease, Hefei, Anhui, China

**Keywords:** Parkinson’s disease, deep brain stimulation, functional connectivity, uric acid, machine learning

## Abstract

**Introduction:**

Parkinson’s disease (PD) is a neurodegenerative disorder characterized by dyskinesia and is closely related to oxidative stress. Uric acid (UA) is a natural antioxidant found in the body. Previous studies have shown that UA has played an important role in the development and development of PD and is an important biomarker. Subthalamic nucleus deep brain stimulation (STN-DBS) is a common treatment for PD.

**Methods:**

Based on resting state function MRI (rs-fMRI), the relationship between UA-related brain function connectivity (FC) and STN-DBS outcomes in PD patients was studied. We use UA and DC values from different brain regions to build the FC characteristics and then use the SVR model to predict the outcome of the operation.

**Results:**

The results show that PD patients with UA-related FCs are closely related to STN-DBS efficacy and can be used to predict prognosis. A machine learning model based on UA-related FC was successfully developed for PD patients.

**Discussion:**

The two biomarkers, UA and rs-fMRI, were combined to predict the prognosis of STN-DBS in treating PD. Neurosurgeons are provided with effective tools to screen the best candidate and predict the prognosis of the patient.

## 1. Introduction

Parkinson’s disease (PD) generally develops between 55 and 65 years of age, affecting 1–2% of people over 60 years of age, or about 0.3% of the total population (Ascherio and Schwarzschild, 2016; Cerri et al., 2019). (UA) is the final product of purine metabolism and is considered an antioxidant in the body. Previous studies have shown that UA inhibits free radical-induced lipid peroxidation and DNA damage, thus acting to protect nerve cells (Narendra et al., 2018; Ya et al., 2018; Mahoney-Sánchez et al., 2021).

Changes in UA levels are associated with several disease states. Abnormally high levels of UA are associated with gout, high blood pressure, cardiovascular disease (Kleber et al., 2015; Gherghina et al., 2022; Méndez-Salazar and Martínez-Nava, 2022). In contrast, lower levels of UA have been confirmed with PD, Alzheimer’s disease (AD), multiple sclerosis (MS) and development of Meg syndrome are associated with (Koch and De Keyser, 2006; Du et al., 2016; Chang and Chen, 2020; Ellmore et al., 2020; van Wamelen et al., 2020; Guan et al., 2021; Seifar et al., 2022). In addition, UA affects the brain structure of PD patients. An MRI using a stationary state function in PD patients (rs-fMRI) found UA levels and broad white matter The integrity of (WM) has a significant correlation (Lee et al., 2020). At the same time, some researchers have shown cortical functional connectivity between UA and PD patients (FC) is closely correlated with high levels of FC in patients with high PD UA and negative correlation with motor symptoms (Lee et al., 2018). These results show that UA is an important biomarker for patients with PD and can be analyzed in combination with rs-fMRI.

Deep brain stimulation (DBS) is becoming one of the most effective treatments for patients with advanced PD, and many previous studies have shown that DBS can significantly improve motor symptoms in patients with PD (Chang et al., 2022; Mei et al., 2022). Interestingly, in PD patients with bilateral subthalamic nucleus (STN) DBS, we observed a positive correlation between UA and postoperative motor symptom improvement. So we guess whether UA can be analyzed in conjunction with rs-fMRI, two biomarkers, to predict the outcome of STN-DBS treatment of PD. The mechanism by which STN-DBS improves motor symptoms in patients is unclear. Some researchers compared the rs-fMRI before and after STN-DBS and found that STN-DBS altered graph theoretical indicators, FC and WM integrity, resulting in significant improvement of motor and mental symptoms in PD patients (Prent et al., 2019; Huang et al., 2022). This suggests that the prognosis of DBS in PD may depend on connectivity between brain regions. Several researchers previously examined structural and functional brain connections associated with PD prognosis after STN-DBS and tested their ability to predict the efficacy of independent cohorts (Horn et al., 2017). Artificial intelligence and machine learning have become increasingly important in healthcare decision-making and prediction in recent years (Naik et al., 2022a,b). Based on the above, we aimed to explore whether FCs associated with UA in PD patients are associated with prognosis in PD treated with STN-DBS, and whether these FCs could be used to predict the improvement of motor symptoms in PD patients treated with STN-DBS. It is hoped that integrated analysis of UA and rs-fMRI can be combined with machine learning to predict the prognosis of STN-DBS treatment in PD patients, so as to provide help for neurosurgeons to predict patients’ conditions and screen patients.

## 2. Participants and methods

### 2.1. Participants

Medical records and questionnaire results were retrospectively collected from patients with PD who underwent STN-DBS at the First Hospital of the University of Science and Technology of China from September 2019 to April 2020. The study protocol was approved by the Ethics Committee of our hospital (2022-RE-154). The included patients had intermediate-to-advanced PD, and the exclusion criteria were moderate/severe cognitive impairment, persistent severe psychiatric disorder, severe atrophy or diffuse ischemic lesions on MRI, and systemic diseases that prevented surgery. Moreover, the medical records of age- and sex-matched healthy participants who underwent annual physical check-ups at the same hospital were collected as healthy controls (HC).

### 2.2. Acquisition clinical assessment

Demographic and clinical variables, including age, sex, duration of illness, and levodopa equivalent dose, were collected from patients’ medical records and questionnaires. Symptom severity was assessed using the Unified Parkinson’s Disease Rating Scale (UPDRS-III). The patients’ motor symptoms were reassessed 2 years after surgery using the UPDRS-III scale during the stimulation and medication on period, and the patients’ UPDRS-III score improvement rate was subsequently calculated. The Hamilton Anxiety (HAMA) and Hamilton Depression (HAMD) scales were used to assess the psychological status of patients. The Montreal Cognitive Assessment (MoCA) and the Mini-Mental State Examination (MMSE) scales were used to assess cognitive status. UA values obtained 5 days before STN-DBS were included in the analysis. Each specimen was assayed by the Department of Clinical Laboratory in 2 h post-collection. To be specific, UA was examined according to liver tests. The above clinical variables were determined by the standard automatic counters.

### 2.3. MRI data and preprocessing

For PD patients and HC, a 3.0 T MR scanner (Discovery MR750; General Electric Healthcare, Chicago, IL, USA) with an eight-channel phased-array head coil was used. Prior to scanning, the researchers placed earplugs in the subjects’ ears to isolate noise. The participants were then instructed to immobilize their heads with sponge pads to reduce unconscious activity. During the scans, the subjects were allowed to close their eyes, but remained awake to avoid specific, intense ideation activities. We explicitly instructed the participants not to fall asleep during the entire scan. We further confirmed that the participants were awake throughout the scan after completion. Functional and structural MRI data were acquired with a 3T GE (Achieva TX) MRI scanner in the OFF medication state before DBS surgery, following an 12-h period of medication withdrawal. Structural images were acquired using a sagittal magnetization prepared rapid gradient echo three-dimensional T1-weighted sequence [repetition time (TR) = 8.5 ms, echo time (TE) = 3.2 ms, inversion time (TI) = 450 ms, and flip angle (FA) = 12°]. Functional MRI images were obtained using the following SE-EPI sequence: repetition time [TR] = 2,000 ms, repetition time [TR] = 30 ms, slice thickness/gap = 3.6/0 mm, axial slices = 38 layers, flip angle [FA] = 90°, FOV = 256 × 256 mm, matrix size = 64 × 64, and scanning time = 484 s.

Data pre-processing was conducted with Resting-State fMRI Data Analysis Toolkit plus V1.25 (RESTplus V1.25),^1^ which is based on Statistical Parametric Mapping (SPM).^2^ Data from 242 volumes were separately acquired as functional scans of the subjects and healthy controls. The first 10 volumes of each functional scan were excluded to correct for subject habituation to the scanning environment and for magnetization stability. Slice-timing correlation was performed to help compensate for differences in acquiring data across all slices with the FOV at any given time point; realignment for head-motion correction was also considered; one healthy control whose head motion exceeded 3.0 mm or involved rotation exceeding 3.0° during the fMRI scanning was excluded. Individual 3D T1-weighted anatomical images were co-registered to the functional images and spatially normalized to the Montreal Neurological Institute template. Each voxel was resampled to 3 mm× 3 mm× 3 mm. Subsequently, the resampled images were smoothed using a 6 mm full-width half-maximum (FWHM) isotropic Gaussian kernel. Subsequently, a linear trend and bandpass filter (0.01∼0.08 Hz) were used to remove the effect of high-frequency noise. Finally, Friston-24 head motion parameters, cerebrospinal fluid signal, white matter, and the Friston- 24 head motion parameters model were considered as nuisance covariates and were regressed from fMRI signals. The resulting data were analyzed further. Subsequently, two voxel-wise whole-brain analytic methods were applied.

### 2.4. DC calculation

To identify functional hubs, The voxel-wise correlation matrix was performed by Pearson’s correlation for whole brain time series. Then we set the correlation coefficients with *r* ≥ 0.25. The threshold was used to eliminate counting voxels that had low temporal correlation. We took each voxel as a node, and the correlation value between any pair of voxels as the internodal edge weight. The weighted DC of each voxel was further divided by the global mean DC of every individual for group comparison.

### 2.5. Spatial correlation analysis of correlations with UA

In this study, the mALFF and mReHo values of each region of interest (ROI) in the AAL3-170 atlas were separately extracted as candidate features. The AAL3-170 atlas (accessed June 4, 2022)^3^ is an improved version of the AAL2 atlas that divides the entire brain into 166 ROI (Supplementary Table 1). In addition, two small regions of the AAL3 atlas (nos. 133–134) were not defined because the original voxel size of 1 mm× 1 mm× 1 mm was resampled to 3 mm× 3 mm× 3 mm; thus, the number of remaining regions in the AAL3-170 atlas was 164. Serum UA values of PD patients were correlated with the mALFF and mReHo values of each of the 164 brain regions. False discovery rate (FDR) correction was not performed for the 164 correlated values, with the threshold set to 0.05.

### 2.6. Functional connectivity analysis

Using the AAL3 template, the DC values and UA significantly correlated with the ROIs were filtered. Following correlation analysis, 15 significantly correlated ROIs remained between DC values and UA. The average resting state blood oxygenation level-dependent (BOLD) time series for each ROI was extracted. The BOLD time series for each ROI was then correlated with the BOLD time series of every other ROI (Pearson’s correlation) for each participant. A 15 × 15 correlation matrix was obtained for each subject. Fisher’s Z transformation was applied to the FC maps for subsequent statistical analysis.

### 2.7. Statistical analyses

Correlations between the UPDRS-III score improvement rate and UA values were analyzed using Pearson’s correlation coefficient test. A two-sample *t*-test was performed in the PD and HC groups to detect zFC differences with FDR correction (*p* < 0.05), with * representing significantly abnormal zFC values between the two groups.

### 2.8. Feature extraction and SVR model training

In the paper, we use SVR to investigate whether inter-group differences in functional connection values can predict the rate of improvement after STN-DBS. Radial Basis Kernel is used in SVR model to find a non-linear regression line and analysis steps. Are done using the LIBSVM software package.^4^ The functional connectivity values of PD group come from differences between groups as features (These ROIs for functional connectivity were selected from significant correlation between DC and UA). The patient’s improvement after STN-DBS as label. Each feature is normalized to between −1 and 1, so do as label. We applied a leave-one-out cross-validation (LOOCV) to train SVR model, and a “grid search” method was used to access parameter optimization. The adaptability of the model was assessed by the Pearson’s correlation coefficient(r) and mean squared error (MSE) between the original and the predicted rate of improvement.

The optimal parameter settings of SVR:

Kernel name: RBF.

Parameter optimization algorithm: Gird search algorithm.

Cross-validation type: Leave one out.

K fold number: 32.

C: 8388608.

g: 2.7387e–07.

p: 0.4.

## 3. Results

### 3.1. Participants’ characteristics

**FIGURE 1:**
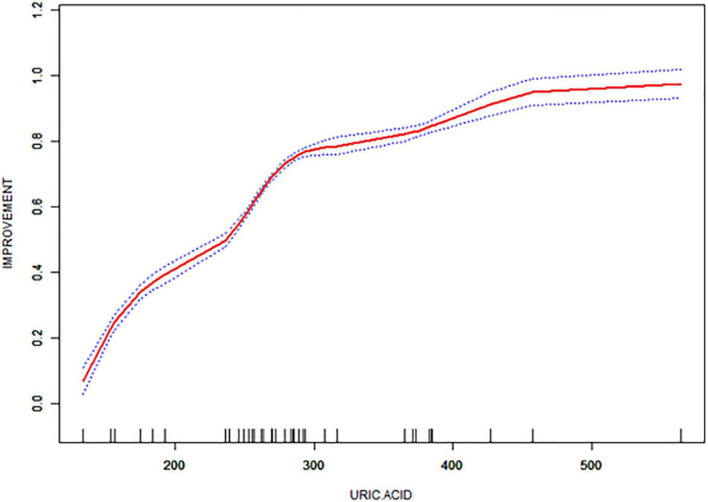
Correlation of UPDRS-III score improvement rate with uric acid (UA) values in the enrolled patients.

Table 1 presents the characteristics of the study participants. Thirty-eight patients were consecutively enrolled, none of whom were lost to follow-up, comprising 17 (44.74%) men and 21 (55.26%) women aged 41–73. The mean improvement rate according to the UPDRS-III score 2 years postoperatively in the medicine-on-period was 66%. The mean preoperative UA level of the patients was 288.45 ± 87.05 μmol/L 5 days prior to STN-DBS. Thirty-two healthy participants were included in the analysis. The median age of the healthy participants was 63 years (range: 44–75 years), and the majority were also female (53.12%). The mean preoperative UA level of the healthy participants was 327.36 ± 10.57 μmol/L. UA values were positively correlated with the improvement rate of the UPDRS-III score two years after surgery (Figure 1, Supplementary Tables 2, 3).

**TABLE 1:** Characteristics of the patients with Parkinson’s disease (PD) and healthy controls (HC).

Variables	PD	HCs	*P-*value
No	38	32	
Age (years, mean ± SD)	58.87 ± 7.61	63.09 ± 1.38	0.225
Gender			0.860
Male	17 (44.74%)	15 (46.87%)	
Female	21 (55.26%)	17 (53.13%)	
Uric acid (μmol/L)	288.45 ± 87.05	327.36 ± 10.57	0.036
LED	642.11 ± 399.51		
Duration (years)	8.84 ± 3.83		
Age of onset (years)	50.03 ± 7.30		
cUPDRS III med off	57.29 ± 12.16		
UPDRS III med on	29.05 ± 10.43		
UPDRS III med off Postop	50.79 ± 18.13		
UPDRS III med on Postop	19.34 ± 11.36		
UPDRS III med on Postop improvement rate	0.66 ± 0.21		
H–Y			
2.5	3 (7.89%)		
3	18 (47.37%)		
4	13 (34.21%)		
5	4 (10.53%)		

### 3.2. Brain connectivity estimation

Six PD subjects were excluded because the structural image data dimensions were not consistent with those of the other subjects. In addition, one of the HC subjects was excluded because of head movement of more than 3.0 mm or 3°. A total of 32 subjects with PD and 31 HCs were included in the final fMRI data analysis.

**FIGURE 2:**
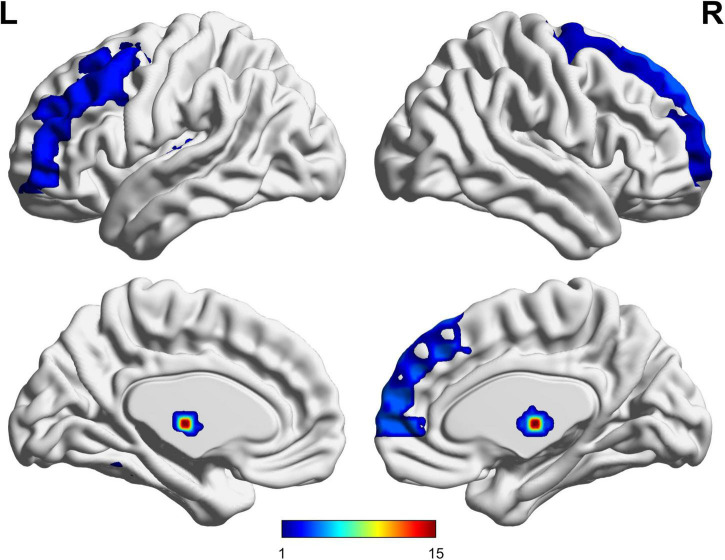
Functional region of interests (ROIs) used in the study.

**FIGURE 3:**
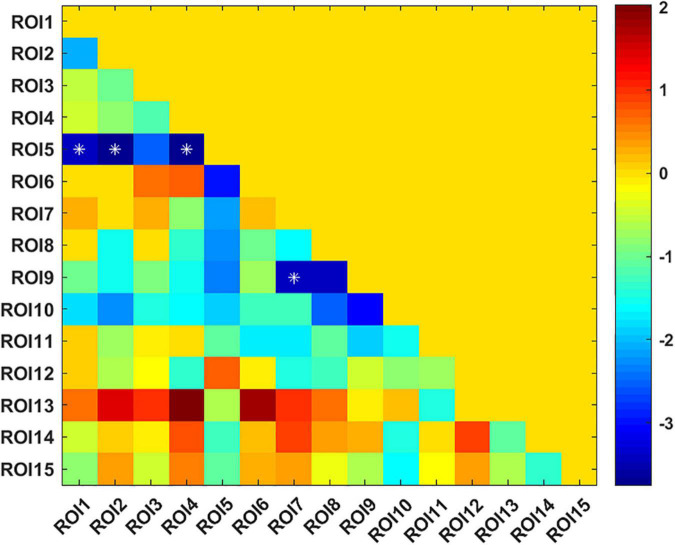
*T*-value matrix of functional connectivity between the Parkinson’s disease (PD) and healthy controls (HC) groups. 4 ROI-pair significant FCs (marked as *) from the lower triangular part of the matrix were retained (redundant elements and diagonal elements were excluded) in a 15 × 15 matrix, namely, the ROIs with significant correlation between DC and uric acid (UA).

**FIGURE 4:**
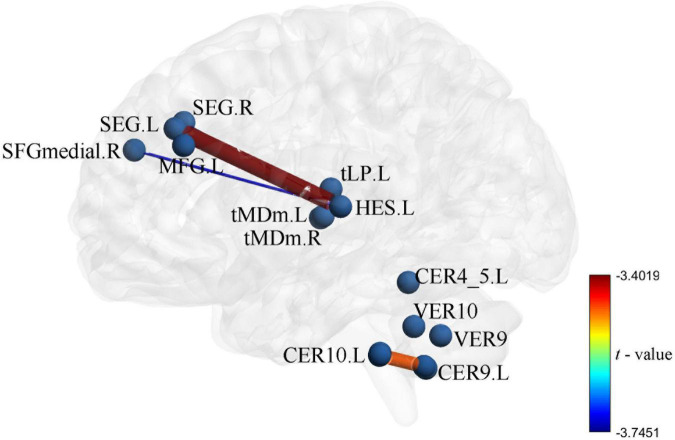
Intergroup differences in functional connectivity between Parkinson’s disease (PD) and healthy controls (HC) groups the zFC pattern of 4 function connectivitys (FCs) between PD and HC, these FCs were significantly correlation in DC and uric acid (UA).

The UA values of PD subjects were correlated with the DC values in 164 brain regions. The 164 correlated values were FDR corrected, DC values were correlated with UA values in 15 brain regions in the Table 2. The 15 brain regions in which the DC values were significantly correlated with UA values were used as ROIs (Figure 2). The zFC matrices were subsequently calculated for each participant by calculating the functional connectivity values of the ROI. A two-sample *t*-test was then performed using the zFC in the PD and HC groups, and the *t*-test results were FDR corrected to *p* < 0.05 to obtain the *t*-value matrix of functional connectivity between the two groups, as shown in Figure 3. 4 ROI-pair FC from the lower triangular part of the matrix were retained (redundant elements and diagonal elements were excluded) in a 15 × 15 matrix, namely, the ROIs with a significant correlation between DC and UA (Figure 3 and Table 3). Intergroup differences in FC between the PD and HC groups are shown in Figure 4.

**TABLE 2:** Region of interests (ROIs) where DC values correlate with uric acid (UA) values.

Index	ROIs	*r*	*P-*value
3	Frontal_Sup_2_L	0.444*	0.011
4	Frontal_Sup_2_R	0.425*	0.015
5	Frontal_Mid_2_L	0.398*	0.024
20	Frontal_Sup_Medial_R	0.394*	0.026
83	Heschl_L	−0.350*	0.049
101	Cerebellum_4_5_L	−0.364*	0.041
109	Cerebellum_9_L	−0.359*	0.044
110	Cerebellum_9_R	−0.431*	0.014
111	Cerebellum_10_L	−0.365*	0.040
112	Cerebellum_10_R	−0.415*	0.018
119	Vermis_9	−0.423*	0.016
120	Vermis_10	−0.368*	0.038
123	Thal_LP_L	−0.369*	0.038
135	Thal_MDm_L	−0.405*	0.021
136	Thal_MDm_R	−0.380*	0.032

*P < 0.05.

**TABLE 3:** Compared with healthy controls (HC) group, there were significant differences in DC-ROI-pair function connectivity (FC).

FC	*t*-value	*P*-value
Frontal_Sup_2_L–Heschl_L	−3.402	0.001
Frontal_Sup_2_R–Heschl_L	−3.745	<0.001
Frontal_Sup_Medial_R–Heschl_L	−3.704	<0.001
Cerebellum_9_L–Cerebellum_10_L	−3.473	<0.001

### 3.3. Prediction and validation of SVR

**FIGURE 5:**
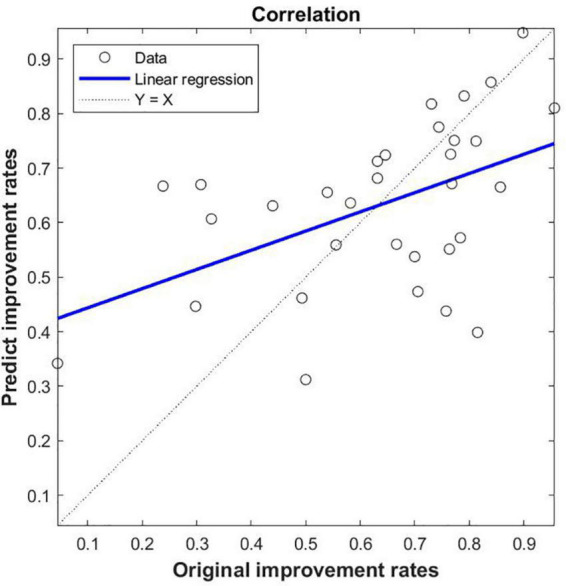
SVR-based prediction of uric acid (UA)-related function connectivity (FC) on the rate of improvement of motor symptoms in patients with Parkinson’s disease (PD) following STN-DBS. The predicted value of SVR was correlated with the original value; the feature of SVR was that the zFC of PD was different between PD and HC, and the ROIs were significantly correlated with DC and UA.

The functional connectivity values of PD groups come from differences between groups as features (These ROIs for functional connectivity were selected from significant correlation between DC and UA), the Pearson’s correlation coefficient was calculated between the actual improvement rate and the predicted improvement rate (*r* = 0.487, *p* < 0.005, MSE = 0.173) (Figure 5). This shows that using the SVR model, serum UA-related differential brain function connectivity in patients with PD can predict the improvement rate of motor symptoms following STN-DBS.

## 4. Discussion

STN-DBS is an accepted treatment for a variety of motor disorders, especially PD. STN-DBS has shown long-term efficacy and has been used in patients with advanced PD for many years (Ashkan et al., 2017). Although the effectiveness of STN-DBS in treating PD is satisfactory, its mechanism needs to be further clarified. Previous studies have found that the prognosis of STN-DBS is associated with brain connectivity. A previous study based on preoperative diffuse tensor imaging (DTI) in patients with PD found that the regions of the brain most associated with the efficacy of STN-DBS include the thalamus, nigra, brainstem and superior frontal gyrus (Vanegas-Arroyave et al., 2016). In addition, functional connectivity can be assessed by the blood oxygen level dependent sequence (BOLD) of rs-fMRI. Many rs-fMRI-based studies have shown that STN-DBS regulates all major components of the motor cortex-striatum-thalamus-cortex loop, including the cortex-striatum, thalamus-cortex, and direct and indirect basal ganglion pathways (Kahan et al., 2014). Also DBS is an expensive and complex treatment. Prior to STN-DBS, doctors conduct a detailed assessment of PD patients to select the most appropriate patient to ensure the best response to DBS. Therefore, some studies attempt to predict results on the basis of brain connections. Some studies have found that ultradirectional, direct and basal intake of STN can predict the clinical status and therapeutic response of DBS (Horn et al., 2017). However, DTI or fMRI images are not always available for various reasons; Therefore, some researchers try to predict the prognosis on the basis of the common connection group. Interestingly, studies have shown that structural and functional connectivity is a predictor of clinical improvement and estimated responses in individual patients, with significant errors (Schlesinger and Schlesinger, 2008). Therefore, predicting the prognosis of STN-DBS based on machine learning synthesis of blood biomarkers and rs-fMRI may be a new direction.

Uric acid has been shown to play an important role as a natural antioxidant in the development and progression of PD. However, the effect of UA on the efficacy of STN-DBS in treatment of PD remains unknown. Interestingly, we observed a positive correlation between UA levels and the rate of improvement in motor symptoms in patients with PD following STN-DBS. Previous studies have similarly demonstrated that low levels of serum UA are involved in the pathogenesis and progression of PD, although its sensitivity as a single biomarker for PD is low (Li et al., 2017; Koros et al., 2021). Similarly, previous studies based on rs-fMRI showed a close relationship between UA and WM and FC integrity in patients with PD. Based on this, we organically combine UA, a biomarker for PD patients, with rs-fMRI and predict the prognosis of STN-DBS by machine learning. DC describes the strength of the brain network connection between an individual protein and all voxels of the whole brain, indicating the importance of this voxel as a network node. We selected DC and serum UA values as characteristics of ROI in PD patients to build FC, using the SVR model for machine learning. Through these explorations, we found that the FCs of Frontal_Sup_2_L–Heschl_L, Frontal_Sup_2_R–Heschl_L, Frontal_Sup_Medial_R–Heschl_L, Cerebellum_9_L–Cerebellum_10_L can predict the prognosis of STN-DBS.

Our research has several novel aspects. First, we identified a set of possible imaging biomarkers prior to STN-DBS treatment to predict the clinical response 1 year after treatment. Therefore, the establishment of pre-operative predictor of therapeutic response has important clinical value, which helps neurosurgeons to predict the efficacy and screen patients before DBS. Secondly, ROI method is widely used in previous research. This approach focuses on selected brain regions, but may omit other brain regions that are critical to the underlying pathophysiology of PD (Gong et al., 2014). In contrast, the DC analysis was used in our study, which used the strength of brain network connections between individual and all voxels of the whole brain. Third, we combine rs-fMRI with UA, a PD blood biomarker, to improve the predictive performance of neuroimaging. Fourth, most previous studies that attempted to determine predictors of therapeutic responses used univariate statistics, which applied to group level predictions (Koros et al., 2021); Instead, SVR analysis, the pattern classification technique used in our study, is a promising individual level prediction tool (Redlich et al., 2016).

In this study, global signal regression and scrubbing were not used to process the data. Previous research has shown that global signal regression can cause reductions in sensitivity and can introduce false deactivations in studies of task activation since the assumption of orthogonality can be violated when the experimentally induced activations contaminate the global signal (Murphy et al., 2009). In additional, discarding problematic volumes (scan “scrubbing”), or alternatively including spike regressors to act as catch-alls for non-linear and non-quadratic spin history effects at problematic time points provides further defense from motion-induced artifacts. However, results have been mixed as to whether any of these participant-level motion correction approaches completely remove inter-individual differences in motion-related MR signal changes (Muschelli et al., 2014).

The present study has several limitations. First, our current study is retrospective and lacks reproducibility analysis (testing the same individual under the same conditions at two different time points). Therefore, it may be a potential confounder of unknown significance. As mentioned above, our study was retrospective; therefore, the required sample size and statistical efficacy were not estimated at this time, and predictive analyses were performed after collection of follow-up information. In addition, we did not collect postoperative fMRI data because of possible artifacts and MRI heating of the implant. Therefore, for safety reasons, we recommend performing long-term follow-up prior to postoperative data collection.

## 5. Conclusion

The results of the present rs-fMRI-based analysis showed that UA-related FCs in patients with PD are closely related to the prognosis of STN-DBS, and can predict the prognosis of STN-DBS by machine learning. Effective tools are provided for neurosurgeons to screen the best patient candidates and to predict patient outcomes.

## Data availability statement

The original contributions presented in this study are included in this article/Supplementary material, further inquiries can be directed to the corresponding author.

## Ethics statement

The studies involving human participants were reviewed and approved by Ethics Committee of the First Affiliated Hospital of USTC. The patients/participants provided their written informed consent to participate in this study.

## Author contributions

BC and CN jointly completed the experiment and the writing. CX, JM, PC, and MJ assisted in the writing and followed up patients. CSN took overall control of the whole study. All authors contributed to the article and approved the submitted version.

## Footnotes

1
http://restfmri.net/forum/index.php

2
https://www.fil.ion.ucl.ac.uk/spm/

3
https://www.oxcns.org/aal3.html

4
https://www.csie.ntu.edu.tw/~cjlin/libsvm/
